# Supplementary material for: Exosome-Mediated Activation of Neuronal Cells Triggered by γ-Aminobutyric Acid (GABA)
Source: Nutrients. 2021 Jul 25;13(8):2544. doi: 10.3390/nu13082544 (PMC8399553; doi:10.3390/nu13082544)
Supplement: Supplementary file 1 [file nutrients-13-02544-s001.zip › nutrients-1263957-supplementary.pdf]

Table S1 Target genes of four miRNA

| | | | | | |
|---|---|---|---|---|---|
| CSMD2 | CYB5RL | LPA | CASC5 | NR2E3 | CD70 |
| CLEC12B | IL24 | TRAPPC3 | PARP11 | ENO4 | LAIR1 |
| PITPNC1 | PRSS22 | BIRC7 | RELL1 | GLP2R | LRRC7 |
| IL21R | SLC22A23 | CHRDL1 | INPP4B | FFAR4 | WDR64 |
| SOST | C6orf132 | RIT1 | KIR2DL4 | EXOC5 | ZNF829 |
| ENPP1 | GRIPAP1 | CTAG2 | PLD5 | 10-Mar | ZNF471 |
| SCN2B | SPG7 | CXCL16 | DOCK9 | PRY2 | PI16 |
| SOX7 | SPTB | IL6R | PLEKHA5 | DIS3L2 | TNN |
| MAMLD1 | CASP10 | SEMA3A | DCBLD1 | HLA-DOB | KAT6B |
| PLA2G6 | NPAS3 | HTRA4 | GRHL3 | HTR1F | CCDC64 |
| NFIA | LEP | SLC5A7 | ASPG | UNKL | SLC12A3 |
| SMPDL3B | CASC4 | FAM26E | SCNN1D | IL12RB2 | EPSTI1 |
| CIT | NFAM1 | C6orf15 | CCDC126 | PCDHA1 | SLC45A2 |
| ZNF589 | GSG1 | GCSAM | IGSF23 | ACSL6 | NEK6 |
| SGPP2 | IRF2 | NRIP1 | SETBP1 | TVP23C-CDRT4 | CDH23 |
| FAM196A | EPB41 | SLC23A3 | KIF18B | AVP | SLC24A1 |
| BRD3 | SCN5A | RNF212 | RHBDL2 | ZNF431 | KLHDC7B |
| PDXK | SHANK2 | EVPLL | EPM2A | ZBTB21 | SP140 |
| SLC6A17 | RHBDD1 | CXCL1 | KCNK13 | MSL3 | RDX |
| HAPLN4 | NME9 | KCNN2 | FRAS1 | FEM1A | DOK7 |
| AKAP4 | SH3RF3 | KLHL30 | ECEL1 | HS6ST3 | DCTN5 |
| MPPED2 | PKD1 | PCSK6 | KRT6A | RETNLB | PCDHA5 |
| ATP2A1 | CLCN6 | SHC4 | PCDHA8 | TRPM3 | RUFY2 |
| OSR1 | C11orf86 | ABHD13 | ZNF320 | SYDE2 | FAM206A |
| TMCC2 | NEUROG1 | SPRR2E | CELF3 | PDE1C | P4HA3 |
| PEX2 | NXPH3 | LDB3 | C22orf24 | PRRG2 | MTCP1 |
| AFF1 | SIGLEC10 | CPXM2 | ARHGAP40 | DEPTOR | CKLF |
| CCL19 | HVCN1 | NPY2R | HRH2 | EIF4E2 | LDLRAD2 |
| ACER2 | SHC3 | DOPEY1 | TET3 | LRCOL1 | NBPF6 |
| FIBCD1 | DUSP16 | ZSCAN1 | CDKN2B | ZDHHC23 | NEDD9 |
| PRDM11 | FGD5 | KRTAP4-1 | SLC9A4 | CCDC144A | |
